# Enhancing CBCT‐based CT synthesis using planning MRI in adaptive proton therapy for head cancer: A deep learning approach

**DOI:** 10.1002/acm2.70367

**Published:** 2025-12-10

**Authors:** Juan Manuel Molina‐Maza, David Viar‐Hernandez, Blanca Rodriguez‐Gonzalez, Javier Sequeiro‐Gonzalez, Juan Antonio Vera‐Sanchez, Alejandro Mazal, Norberto Malpica, Angel Torrado‐Carvajal, Juan Maria Perez‐Moreno, Borja Rodriguez‐Vila

**Affiliations:** ^1^ Universidad Rey Juan Carlos Medical Image Analysis and Biometry Laboratory Madrid Spain; ^2^ Centro de Protonterapia Quironsalud Servicio de Física Médica Madrid Spain

**Keywords:** adaptive protontherapy, deep learning, dual‐energy computed tomography, magnetic resonance image, medical image synthesis

## Abstract

**Background:**

Proton therapy (PT) is recognized as a superior treatment for head cancer (HC) due to its precision and minimal damage to surrounding healthy tissues, relying on computed tomography (CT) data for dose calculations. Adaptive proton therapy (APT) is crucial to address changes in patient anatomy during treatment and update dose accuracy. However, in‐room cone‐beam CT (CBCT) assistance is limited to assessing patient setup, with occasional constraints due to artifacts and/or lower image quality and resolution compared to a CT scan.

**Purpose:**

Although deep learning (DL) techniques can successfully convert a CBCT into a synthetic CT (sCT), soft tissue delineation remains a challenging task. We hypothesized that, by including Magnetic Resonance Image (MRI) in CBCT‐based CT synthesis, the sCT generation could more closely approximate the CT ground truth while improving tissue definition and dose calculation in PT treatment planning.

**Methods:**

We propose a Pix2Pix‐conditional generative adversarial network (cGAN) to synthesize a CT scan by combining two different input images: CBCT and T1‐weighted MRI. ResUnet and SwinUnet were evaluated as the cGAN generator. Additionally, a CBCT‐only‐based CycleGAN was tested.

**Results:**

Model performance improved with the inclusion of MRI data, especially in recovering soft tissue details like eyes and ventricles, with ResUnet models outperforming SwinUnet models. Our cGAN outperformed both the self‐autoencoder approaches and the CycleGAN model. Pix2Pix‐ResUnet (MR‐based) significantly reduced average HU errors in volumes of interest and also enhanced the precision in dose values, as demonstrated in dose differences and profiles.

**Conclusions:**

We demonstrated the promising contribution of MRI to CBCT‐based CT synthesis, enhancing sCT image quality and dose calculation accuracy. Future efforts should aim to collect a larger dataset and validate the integration of MRI in APT.

## 1 INTRODUCTION

One of the most innovative and efficient treatments for head cancer (HC) is proton therapy (PT), a sort of radiotherapy (RT) that employs protons instead of photons to irradiate the patient's tumor. The use of protons for treatment is of medical interest due to its inherent increase in precision and, thus, the reduction in unwanted radiation of healthy tissue surrounding the tumor compared to conventional RT.^1^ The aggressive reduction of head tumors during photon therapy treatment, along with the surrounding critical healthy anatomy, makes PT the ideal treatment due to its superior beam control achieved through the minimal lateral spread and a sharp dose drop‐off, which leads to lower radiation exposure for surrounding healthy tissues, enhanced protection of vital organs, and reduced side effects for patients.^2^ Unlike traditional RT, where dose distribution remains almost consistent throughout the treatment course, anatomical changes represent a key challenge during PT treatment, as they can particularly induce distortions in the dose planned compared to the dose delivered and worsen the treatment accuracy. This is due to the finite range of protons in tissue, as they directly depend on the Bragg peak's position, and even minor anatomical changes, such as patient weight loss or tumor shrinkage, can handicap the dose distribution and cause them to deposit their energy in unintended locations.

A computerized tomography (CT) scan is required to perform dose calculation during PT treatment planning and simulate the dose delivered to the patient tissues.^3^ Such dose estimation is computed using the stopping power ratio (SPR) of every tissue, usually calculated from the CT Hounsfield units (HU). The uncertainty related to the conversion from HU to SPR values makes dose calculation a complex process, as it influences its accuracy.^2^ The use of in‐room cone‐beam computed tomography (CBCT) imaging in the PT workflow, crucial for daily patient positioning and verification of interfractional changes in anatomy, provides a huge dataset that may be employed to calculate dose distribution and decide plan adaptation. Nevertheless, CBCT has several drawbacks compared to conventional CT scans that limit its use to directly estimate dose delivery. The primary limitation of CBCT in the context of adaptive proton therapy (APT) is its inherent inaccuracy in HU compared to the precision offered by conventional CT. Additionally, CBCT is associated with several other disadvantages, including inferior image quality and resolution, various imaging artifacts, and a reduced field of view (FOV). These limitations even increase the uncertainty of HU‐SPR conversion and make CBCT unreliable for dose calculation in APT, necessitating subsequent transformations before using it for dose calculation and treatment planning.^2^, ^4^, ^5^ Moreover, unwanted variations like beam deviations or patient positioning and the presence of noise and artifacts within planning volumes can all negatively impact the accuracy of treatment planning. For all these reasons, APT must be assured to guarantee a precise and effective treatment.^6^, ^7^

In our previous work,^8^ we developed a deep learning (DL) model based on vision transformers (VT) to generate a synthetic CT (sCT) from CBCT head and neck (H&N) images, where those sCTs provided accurate dose calculation for most of H&N tissues when compared with CT ground‐truth results, and the current study represents a continuation of that line of research. Within the PT treatment pipeline, an initial magnetic resonance image (MRI) is requested to precisely delineate the tumor and the surrounding organs at risk (OARs). Once the PT treatment is planned and volumes of interest (VOIs) are delimited, an H&N CT is acquired to compute the optimal dose, and a CBCT is taken ahead of the session to correctly position the patient. Paired CT and CBCT acquisitions are repeated on a weekly basis. Since MRI data provides specific information related to soft tissues, we hypothesize that MRI integration into CBCT‐based CT synthesis could upgrade the delineation and definition of anatomical structures and soft tissue recreation in the sCT, as well as improve the accuracy of SPR values and dose calculation. The brain and skull base regions offer an opportunity to assess the impact of varying tissue densities (such as bone, muscle, and air cavities) with differing heterogeneity that may evolve throughout the treatment course and are highly reproducible in both image sets of pairs, CBCT‐CT. This anatomical area exhibits reduced susceptibility to anatomical and postural variations between CT and CBCT image acquisitions, thereby ensuring superior reproducibility in patient setup. As such, any difference between dose calculation in CT and in sCT will arise from the performance of the sCT generation process. These factors facilitate a focused evaluation of sCT performance in terms of accuracy and uncertainty dose calculation. This work has two major objectives. First, to design and train a Pix2Pix conditional generative adversarial network (cGAN) for generating an sCT using CBCT‐CT pairs that correspond to the multi‐fractionated treatment of the PT patients, along with the planning MRI acquired at the starting point of the treatment. CBCT and MRI were jointly used as the network input, and CT as the network label. Second, assess dose calculation uncertainties in PT on sCT images generated with the trained network.

## 2 STATE‐OF‐THE‐ART

Numerous DL algorithms, both paired and unpaired implementations, were developed over the past years to convert either CBCT or MRI into sCT images for adaptive radiotherapy (ART) or APT planning. With regard to CBCT‐based CT synthesis, some works developed U‐Nets‐based models.^2^, ^9^, ^10^, ^11^ CBCT and planning CT were both employed to generate the replanning CT.^9^ Other works have focused on GANs; in particular, cycle‐consistent GANs (CycleGANs) algorithms have been commonly proposed.^12^, ^13^, ^14^, ^15^, ^16^, ^17^, ^18^, ^19^ A 2D CycleGAN was independently trained and tested on different anatomic regions (H&N, thorax, and pelvis),^15^ and a single CycleGAN was trained with three different anatomical regions (H&N, breast, and lung).^16^ cGANs have also been suggested in other works. For instance, pelvic^20^ and H&N^21^ data were used to develop Pix2Pix cGANs and perform dose evaluation for ART, providing better results than CycleGAN and Unet approaches. Dosimetric evaluation with sCT obtained with a Unet‐based cGAN showed considerable accuracy for planning target volumes and OARs in ART.^22^ Considering MRI‐only RT and PT, several works have been made as well. The use of multiple Dixon images showed acceptable performance when generating human pelvis CT scans with a Unet.^23^ Two‐dimensional cGANs have also been broadly implemented.^24^, ^25^, ^26^, ^27^, ^28^, ^29^ Dixon water, fat, in‐phase MRI pelvis sequences^25^ and multiple MRI abdominal sequences^26^ were tested. Using T1 and T2 sequences simultaneously provided better results when compared with the single sequence approach.^24^ T1 and T2 MRI head acquisitions were used for nasopharyngeal carcinoma,^27^ and T1, T2, and FLAIR MRI sequences for brain radiotherapy.^28^ Photon and proton dose calculations were performed to evaluate pediatric brain sCT obtained training single T1 MRI.^29^ Cycle‐GANs have been applied in ART for unpaired brain and abdomen MRI,^30^ pelvic T2 MRI,^31^ and for H&N images using a multi‐CycleGAN.^32^

We gathered throughout this section various DL approaches for sCT generation in RT and PT, which used either CBCT or MRI inputs, but, to the best of our knowledge, no study was found in the literature based on CBCT and MRI inputs simultaneously. Indeed, the present work represents a notable novelty within the current state‐of‐the‐art on DL techniques for CBCT‐based CT synthesis in APT, and it poses a significant contribution to the medical physics community.

## 3 DATASET

Originally, our initial retrospective dataset comprised 60 patients. However, we needed to specifically collect three different types of images for each patient: a specific T1 MRI sequence, a CBCT, and a CT scan. Since PT is a relatively novel technique and we specifically focus on HC, we needed to narrow down the initial number of patients available. Thus, our final data set contains 137 triple groups of MRI‐CBCT‐CT volumes derived from 27 patients (average age of 30.89 ± 23.05 years, age range of 3–75 years, 11 women) that were retrospectively collected from Centro de Protonterapia Quironsalud (CPT). Our selection criteria encompassed patients diagnosed with brain cancer and treated with PT. Head CBCT and CT scans comprised a five‐day timeframe and the same immobilization, while MRI scans were acquired at the beginning of the PT treatment. CT scans were acquired with a general electric (GE) revolution CT scanner (General Electric Healthcare, Chicago, IL, USA): ASIR‐60 iterative reconstruction, 120 kVp, pixel size = 0.625 mm, slice thickness = 1.250 mm, FOV = 50 cm, matrix size = 512 × 512, metal artifact reduction filters. Besides, in‐room CBCT scans were performed by IBA (Ion Beam Applications, Louvain‐La‐Neuve, Belgium): 100 kVp, pixel size = 0.5371 mm, slice thickness = 1 mm, FOV = 30 cm, matrix size = 512 × 512. The acquisition of CT and CBCT images involves specific head protocols to improve contrast and noise level. The usual quality control is performed on any diagnostic imaging equipment (CT), along with the QA recommendations for CBCT as outlined in TG142.^33^ Additionally, in the CT, QA of the HU‐density curve is conducted weekly using a phantom with inserts of different densities/materials. In the case of MRI, anatomical T1‐weighted volumes were acquired on a GE Signa Premier 3T MRI scanner located at Hospital Universitario Quirónsalud Madrid (Pozuelo de Alarcón, Madrid) using a 32‐channel head coil and an FSPGR sequence with TR = 9.24 s, TE = 3.42 ms, FlipAngle = 10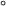, 1 mm isotropic voxel, matrix size = 512 × 512. Our data were retrospectively obtained from a clinical investigation carried out at the CPT, following ethical clearance granted by the local Institutional Review Board and precise adherence to the ethical guidelines of the World Medical Association's Declaration of Helsinki.

DICOM original volumes were first converted to NIFTI format at the beginning of the preprocessing phase. Then, to ensure alignment between the CBCT‐MRI volumes and their corresponding CT volumes, we obtained the rigid registration transformation matrix with RayStation Software (RaySearch Laboratories, Stockholm, Sweden). This matrix was used to map MRI and CBCT volumes, respectively, into CT space with 3D Slicer BRAINs Resample module.^34^ We bounded the HU values to a limited range [‐1024, 3072] in both CBCT and CT images, and, finally, pixel values were min‐max normalized [‐1, 1] for CBCT, CT, and MRI.

## 4 METHODS

### 4.1 Neural network architectures

In this study, we employed four distinct neural network architectures to synthesize CT images: ResUnet (Appendix A.1), SwinUnet (Appendix A.2), Pix2Pix conditional generative adversarial network (GAN) (Appendix A.3), and CycleGAN (Appendix A.4). ResUnet, characterized by its residual connections, enhances feature retention across encoding and decoding stages, thereby preserving spatial details crucial for accurate synthesis. SwinUnet integrates convolutional and transformer components, utilizing self‐attention mechanisms to capture global context and facilitate effective cross‐modality feature extraction. To assess the advantages of adversarial training over standard autoencoding for this synthesis task, both ResUnet and SwinUnet were employed as autoencoders and as generator models within the Pix2Pix GAN framework to generate accurate CT images based on CBCT and MRI inputs.

Additionally, we trained a CycleGAN to synthesize CT images from CBCT data without MRI inputs. CycleGAN employs two generators for bidirectional mappings and two discriminators to evaluate image quality. The training process is driven by adversarial loss, ensuring that the generated images closely resemble those in the target domain, and cycle consistency loss, which enforces coherence by requiring translations from one domain to another and back again to reconstruct the original input. By comparing this CycleGAN approach versus the Pix2Pix GAN, the latter including MRI as input for CBCT‐to‐CT synthesis, our objective was to evaluate the impact of incorporating additional modalities, such as MRI, on the quality of the sCT images. This comparison provides a comprehensive analysis of both methods under varying conditions. Detailed specifications of our methodologies are provided in the Appendix Section A, A.1, A.2, A.3, A.4.

### 4.2 Experiments

Patients were randomly distributed in training (∼75%), validation (∼10%), and test (∼15%) subsets, ensuring that each patient is only in one of the three subsets. The batch size value was set to three slices, kernel weights were randomly initialized, and Adam optimizer was used (learning rate = $1\times 10^{-4}$) to train our models for 100 epochs. Mean absolute error (MAE) was set as the loss function for self‐autoencoder‐based models, Equation (A.3) was set as the loss function for the Pix2Pix cGAN‐based models, and Equation (A.6) was the main loss function for our CycleGAN. From the early epochs, the generator loss function increased exponentially while the error of the discriminator tended to zero quickly, so it became impossible for the GAN model to converge. We solved this issue by using the generator pre‐trained beforehand, and a lower value was also set to the learning rate of the discriminator ($1\times 10^{-6}$) compared to the learning rate of the generator ($1\times 10^{-4}$). Nine experiments were carried out in total: four of them used CBCT and MRI jointly, while the remaining five only used CBCT as input to assess the impact of MRI afterward. ResUnet and SwinUnet were trained first as autoencoders themselves and secondly as the GAN generator. All models were designed with the Tensorflow 2.12 Python library and trained using an NVIDIA A100 80 GB GPU. Official code templates from Pix2Pix‐GAN ^35^, CycleGAN,^36^ and Swin Transformer ^37^ were taken as a basis to precisely build and design our DL models. A compilation of the parameters and hyperparameters of the models is shown in Table 1.

**TABLE 1:** Summary of parameters and hyperparameters for our different architectures.

Architectures	Parameters	Embedding dim/Num filters	Heads	ST/Conv blocks
ResUnet (w/o MRI)	124M	[64,128,256,512,1024]	—	‐ / 30
ResUnet (w/ MRI)	124M	[64,128,256,512,1024]	—	‐ / 40
SwinUnet (w/o MRI)	122M	[128,256,512,1024,2048]	[8,16,32,32,32]	24 / 5
SwinUnet (w/ MRI)	126M	[128,256,512,1024,2048]	[8,16,32,32,32]	31 / 5
Discriminator	5M	[64,128,256,512]	—	‐ / 4

*Note*: Autoencoder/Generator architectures (first four rows on the table) have approximately the same number of parameters in order to be fairly compared.

### 4.3 Clinical and dosimetric evaluation

Delineating OARs with accuracy is vital in PT to avoid damaging healthy tissues and achieve effective treatment outcomes. Therefore, we aim to show statistical evidence that demonstrates the potential improvements in OAR delineation for the head and brain organs when incorporating MRI in CBCT‐based CT synthesis. For this purpose, we employed a widely recognized self‐configuring benchmark for medical image segmentation named Total Segmentator^38^ to specifically assess the segmentation accuracy on some head glands, cavities, and brain structures. Total Segmentator is an external open‐source tool that consists of a DL architecture based on the nnUNet framework.^39^ The delineations obtained with the original CT volumes were taken as the ground truth. Two different metrics were utilized for evaluating the results of OAR delineation: Dice similarity coefficient (DSC) and Hausdorff distance (HD).^40^, ^41^ Each of these two metrics complements the other to provide the robust assessment necessary for OAR delineation in PT. A detailed explanation of each metric can be found in the Appendix Section A.5.

For further evaluation of the impact on the dose distribution, in addition to dose calculation assessment and dose volume histograms (DVH) analysis, a single‐energy lateral beam (sharing the same isocenter as the treatment plan) was used to evaluate the range in each imaging study of the test dataset. A uniform spot spacing of 2.5 mm was applied, covering a square field size between 80 and 100 mm. The field size was selected to avoid tangential irradiation of the patient's surface, and the beam energy was adjusted so that the distal edge extended beyond the midline but remained within the patient's anatomy. A dose grid with 1 mm resolution was used to evaluate 1 × 1 ${\mathrm{mm}}^{2}$ dose lines along the patient's anatomy in the beam direction, allowing the extraction of distal R80 values. R80 is a critical analysis parameter in PT, as it represents the depth at which the delivered dose falls to 80% of its peak value. Accurate estimation of R80 is essential for ensuring precise tumor coverage while avoiding excessive irradiation of surrounding healthy tissues. Each individual dose line was normalized to its maximum value at the Bragg peak position. The resulting 2D R80 matrix from the dose calculations on the actual control CT served as a reference to compare the differences with the R80 values derived from the synthetic CTs. Differences in millimeters were normalized to the R80 in water for the selected energy, as specified in the beam data library (BDL).

## 5 RESULTS AND EVALUATION

### 5.1 Qualitative evaluation

**FIGURE 1:**
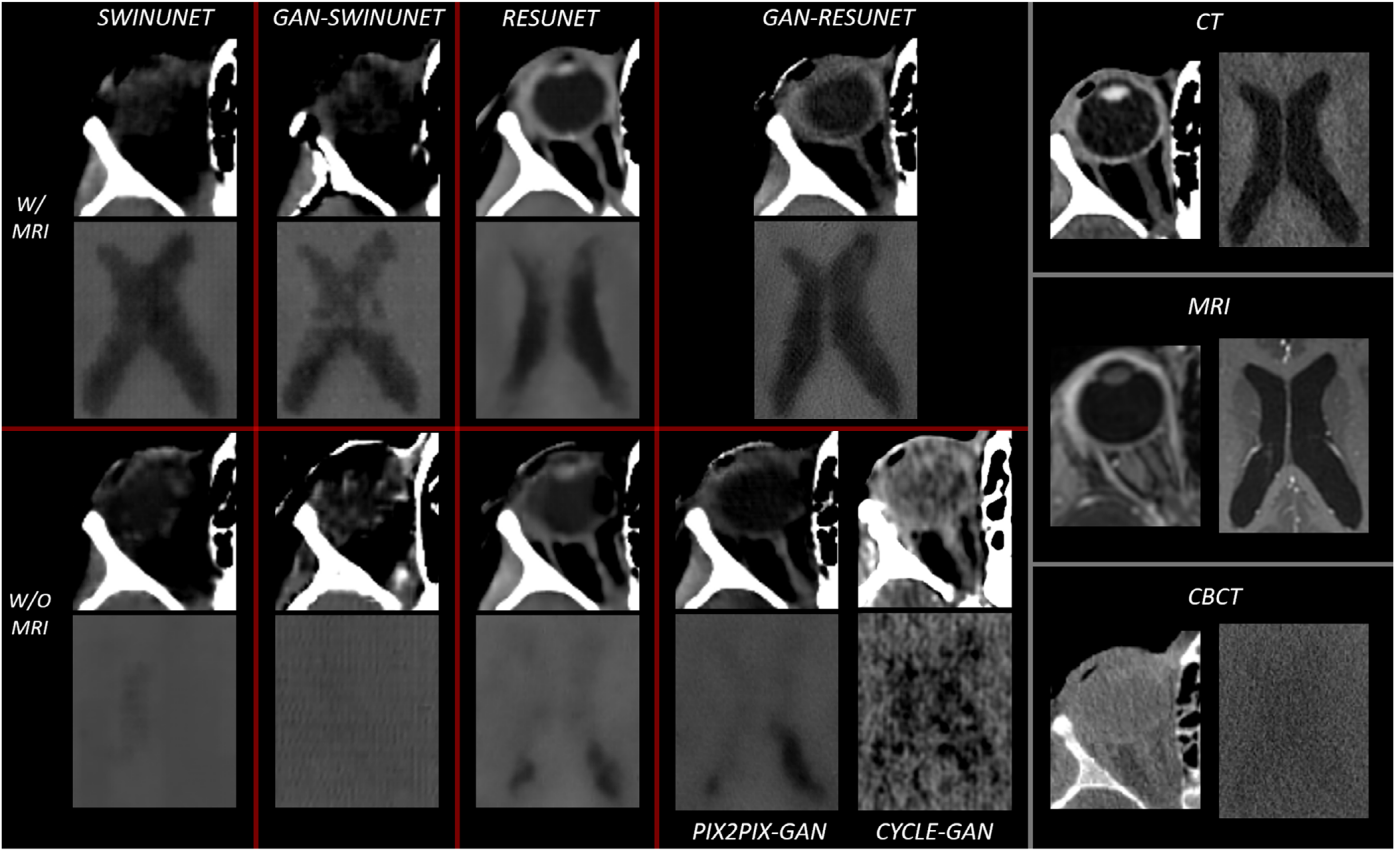
Qualitative visualization for eyes and ventricles in a specific slice. Window width (100 HU) and level (50 HU) are adjusted to facilitate soft tissue visualization. Our nine sCTs related to the different models are presented along with the original CBCT, CT, and MR images. CT, computed tomography; CBCT, cone‐beam CT; HU, Hounsfield units; sCTs, synthetic CTs.

We first focused on assessing the image quality regarding several soft tissues (Figure 1). Optic nerves and eyes are better recovered when including MRI data in both ResUnet and GAN‐ResUnet models, but the latter does not manage to recover the eyes' lens. Our SwinUnet‐based models are incapable of revealing either eyes or optic nerves, no matter whether MRI data is used or not, so they can be discarded for diagnostic and further evaluation purposes. For any model, achieving a good recovery of soft tissue detail and definition in ventricles requires the essential contribution of MRI. Our GAN‐ResUnet outperforms the rest of the models in this region, even though our SwinUnet‐based models yield similar results when compared to those of the eyes and optic nerves. Visual inspection of brain tissues for the CycleGAN model reveals that image quality decreases considerably, particularly for the ventricles. Regarding the eyes, while the definition and detail are poor and appear very blurred, the contours are recovered acceptably. Since the qualitative results of SwinUnet‐based models were not of sufficient quality to be considered applicable in the APT workflow, only ResUnet‐based models have been evaluated more extensively from this point onward.

**FIGURE 2:**
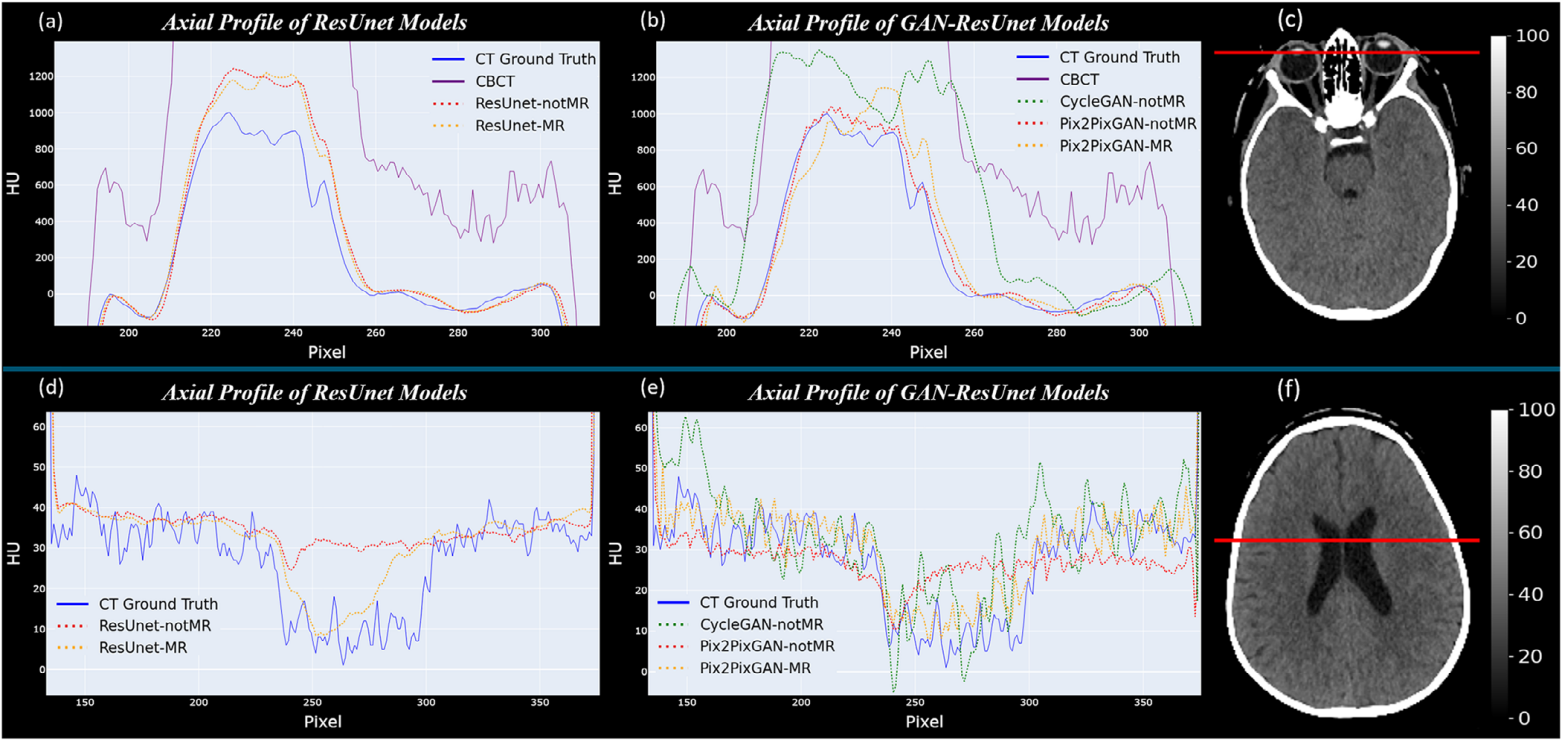
Line profile from an axial slice of a patient. Four charts are displayed in total: charts a,b show the profile in detail for the eyes and eyes' lens region, while charts d,e show it for the ventricles region. ResUnet models (a,d) and GAN‐ResUnet models (b,e) are compared (both MR and not MR‐based approaches) with the CT reference and CBCT profile. The profile is depicted as a red horizontal line on two CT ground truth slices: the eyes slice (c) and the ventricles slice (f). Window width (100 HU) and level (50 HU) are adjusted to facilitate the visualization of the profile on the target soft tissues. CT, computed tomography; CBCT, cone‐beam CT; HU, Hounsfield units; GAN, generative adversarial network.

An axial profile is shown for a slice containing some characteristic soft‐liquid tissue, like the brain ventricles and the eyes, to assess our ResUnet and GAN‐ResUnet models' performance (Figure 2). There is a high inaccuracy of CBCT profile relative to the CT ground truth, both in the sharp peaks as well as within the less abrupt areas corresponding to soft tissue. It becomes even clearer for ventricle plots (Figure 2d,e), where the HU precision of CBCT is so far from the CT ground truth that the profile of the former cannot be displayed in those two plots. The ResUnet network, due to the exclusive use of convolutions for learning, presents a larger smoothing along the profile compared to all GAN‐ResUnet implementations (CycleGAN and Pix2Pix‐GAN). This evidence that the GAN competition between the discriminator and the generator avoids image smoothing and allows keeping the original abrupt HU peaks present along the original CT profile.

For the ventricles region (Figure 2d,e), only MR‐based models can accurately approximate the original *valley* generated by the CT profile. GAN‐ResUnet‐based models better approach the ground truth profile as they maintain reasonably well the amplitude and the frequency of the original CT peaks. However, it is our Pix2Pix‐GAN (MR‐based) that clearly outperforms the rest of the GAN‐based models. Even though the original CT peaks are recovered to some extent with the Pix2Pix‐GAN (non‐MR‐based), the original peak amplitude and HU accuracy were recovered when adding the MRI to the model. In the case of the CycleGAN (not MR‐based), it faithfully follows the average line of the CT ground truth profile, but the amplitude of most peaks is too large and inaccurate, which makes sense with its blurry and noisy image quality shown in Figure 1. In the ocular region (Figure 2a,b), no significant improvement is observed when comparing each MR‐based model with its non‐MR‐based counterpart. Comparing GAN‐based and non‐GAN‐based models, the former come closer to the ground truth. Similar to the ventricles region, the CycleGAN clearly underperformed all Pix2Pix‐GAN‐based models in the eye area.

### 5.2 Quantitative evaluation

**FIGURE 3:**
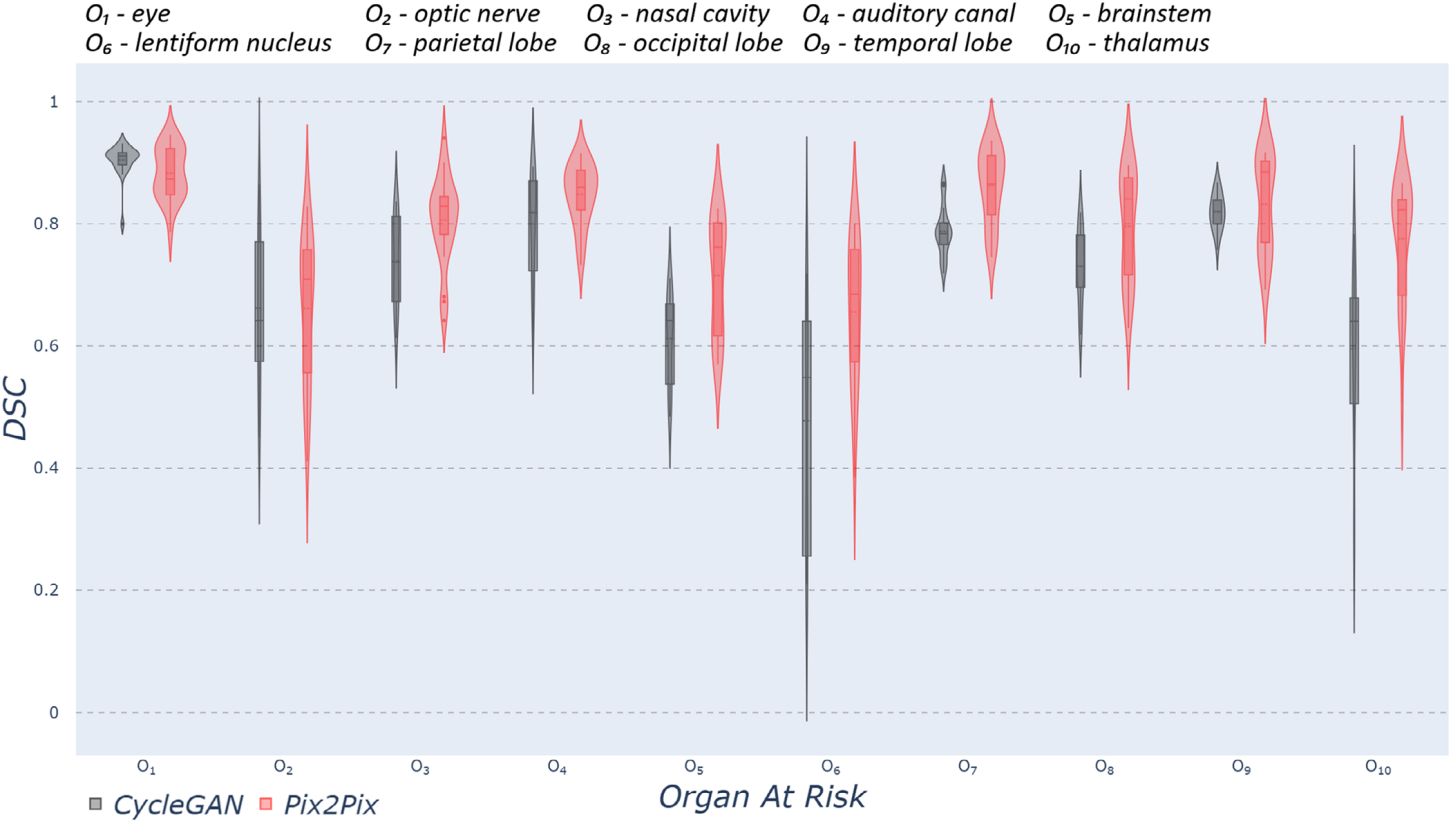
Violin plots for DSC metric comparing two different methods: non‐MR based CycleGAN and MR‐based Pix2Pix within ten different head and brain organs. Each violin plot corresponds to one specific organ, and it is calculated from test subjects. CycleGAV, cycle‐consistent generative adversarial network; DSC, Dice similarity coefficient.

**FIGURE 4:**
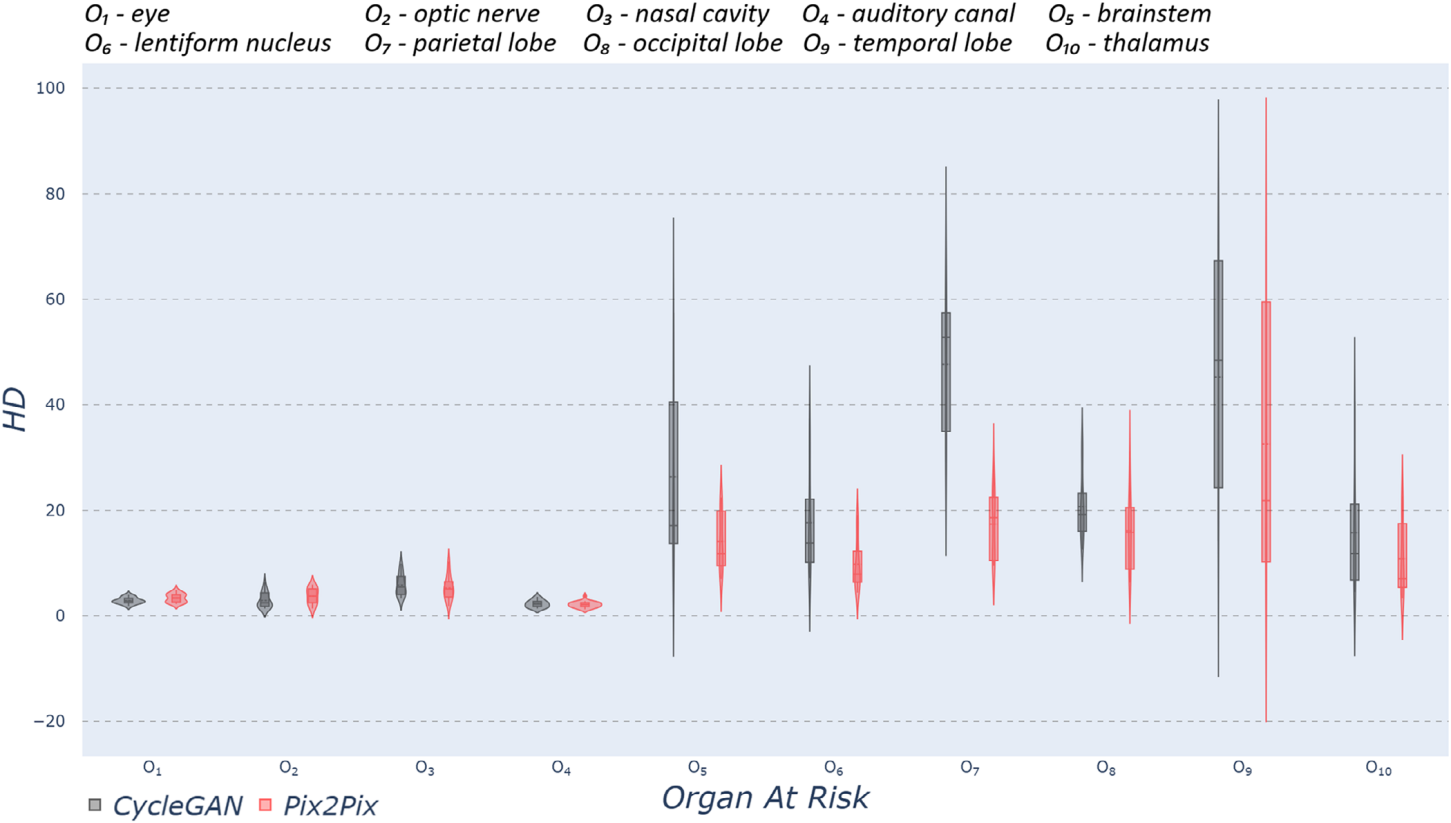
Violin plots for HD metric comparing two different methods: non‐MR‐based CycleGAN and MR‐based Pix2Pix within ten different head and brain organs. Each violin plot corresponds to one specific organ, and it is calculated from test subjects. CycleGAV, cycle‐consistent generative adversarial network; HD, Hausdorff distance; HU, Hounsfield units.

Regarding OARs delineation performance (using DSC, HD), non‐MR‐based CycleGAN significantly outperformed all proposed models except our MR‐based Pix2Pix‐GAN. Thus, for clarity purposes, from now on, we show only the results for the two models mentioned. Figure 3 shows the DSC values, and Figure 4 presents the HD values, only for those head anatomical structures that show statistically significant differences ($p^{\prime }s<0.05$). The rest of the head structures available in Total Segmentator did not show statistically significant differences. In the case of the HD metric, the distribution shows greater variation across organs compared to the distribution of DSC values. Except for the eye and the optic nerve, the remaining structures exhibit higher DSC and lower HD values using Pix2Pix‐GAN (w/MRI) compared to CycleGAN (w/o MRI), although the concentration of value distribution around the mean varies depending on the organ. Some factors may explain why the eyes are a notable exception where the MR‐based model achieves slightly lower DSC values. The eyes are small, compact structures occupying a few voxels, which makes the DSC particularly sensitive to even minimal contour shifts, whereas larger organs tolerate similar deviations without significant metric impact, and the high‐contrast sclera–fat interface of the ocular anatomy might make the segmentation especially sensitive to small intensity or geometry changes. Additionally, Pix2Pix's adversarial training might smooth sharp intensity transitions, while CycleGAN's cycle‐consistency loss preserves the eyes' original geometry more faithfully, despite the model producing lower overall visual quality.

Table 2 presents the quantitative performance of our main models along with CBCT results for comparison, using four different quantitative metrics: MAE, peak signal‐to‐noise ratio (PSNR), structural similarity index measure (SSIM), and relative error (RE) of SPR maps. By including the MRI in the synthesis scheme, we strongly upgrade the model performance. Both sCT models outperformed all CBCT metrics. The model had a statistically significant influence on all metrics as evidenced by the ANOVA test (p‐value < 1%). The Wilcoxon test showed statistically significant differences among all models for each metric ($p^{\prime }s<0.01$).

**TABLE 2:** Quantitative metrics.

Models	MAE (HU)	PSNR (dB)	SSIM	SPR (%)
CBCT	112.384 ± 48.537	24.673 ± 4.106	0.845 ± 0.060	—
CycleGAN‐ResUnet (w/o MRI)	110.792 ± 14.032	25.183 ± 1.028	0.938 ± 0.018	7.008 ± 0.881
**Pix2Pix GAN‐ResUnet (w/ MRI)**	**82.681** ± **13.823**	**27.295** ± **1.549**	**0.956** ± **0.016**	**5.773** ± **0.983**

*Note*: Each of the four metrics was calculated only over the brain tissue, but not over the background image. Mean and standard deviation were calculated over all test subjects.

**FIGURE 5:**
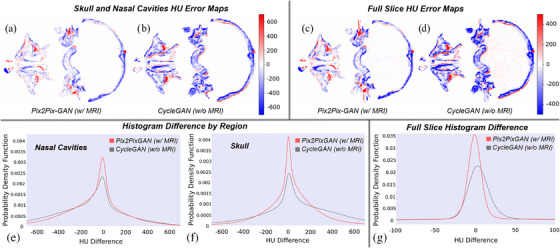
Error maps for HU difference are shown for skull and nasal cavities (a,b) and for all CT tissues (c,d) over a slice from a test patient. Histograms of HU differences were calculated over all test patients and were displayed regarding the nasal cavities (e), skull (f), and all CT tissues (g). Control CT worked as the ground truth reference for being compared with sCTs: MR‐based Pix2Pix‐GAN and not MR‐based CycleGAN. CT, computed tomography; CycleGAV, cycle‐consistent generative adversarial network; HU, Hounsfield units; sCTs, synthetic CTs.

As anatomical changes often happen throughout the treatment, modifying HU values (e.g., enlargement or reduction of a cystic component, paranasal sinuses filling/emptying, etc.), we evaluated the visual distribution and the difference histogram regarding HU error maps specifically over the skull and nasal cavities regions (where MRI does not offer appropriate information) (Figure 5a,b), but also over the whole CT image (Figure 5c,d). Within HU error maps, MRI usage substantially reduces the error intensity, no matter the region analyzed. The errors are predominantly localized around the anatomical boundaries, which enables better recovery of structural details and provides additional anatomical guidance. In contrast, our CBCT‐only‐based CycleGAN model exhibits more widespread and higher errors, particularly around complex regions such as bony structures and soft‐tissue interfaces, worsening the ability to infer nuanced anatomical information. These discrepancies are further reflected in the HU histogram difference calculated over all test patients for nasal cavities (Figure 5e), skull (Figure 5f), and the whole CT (Figure 5g). Our MR‐based model demonstrates a higher fidelity in reproducing HU values, showing a narrower and more centered histogram distribution as opposed to the non‐MR‐based model, the latter showing a broader distribution with a greater deviation with respect to the zero difference value. The central peak is more prominent around zero HU difference for our MR‐based model, indicating a reduced error since it is more concentrated around the zero difference value.

### 5.3 Dosimetric evaluation

**FIGURE 6:**
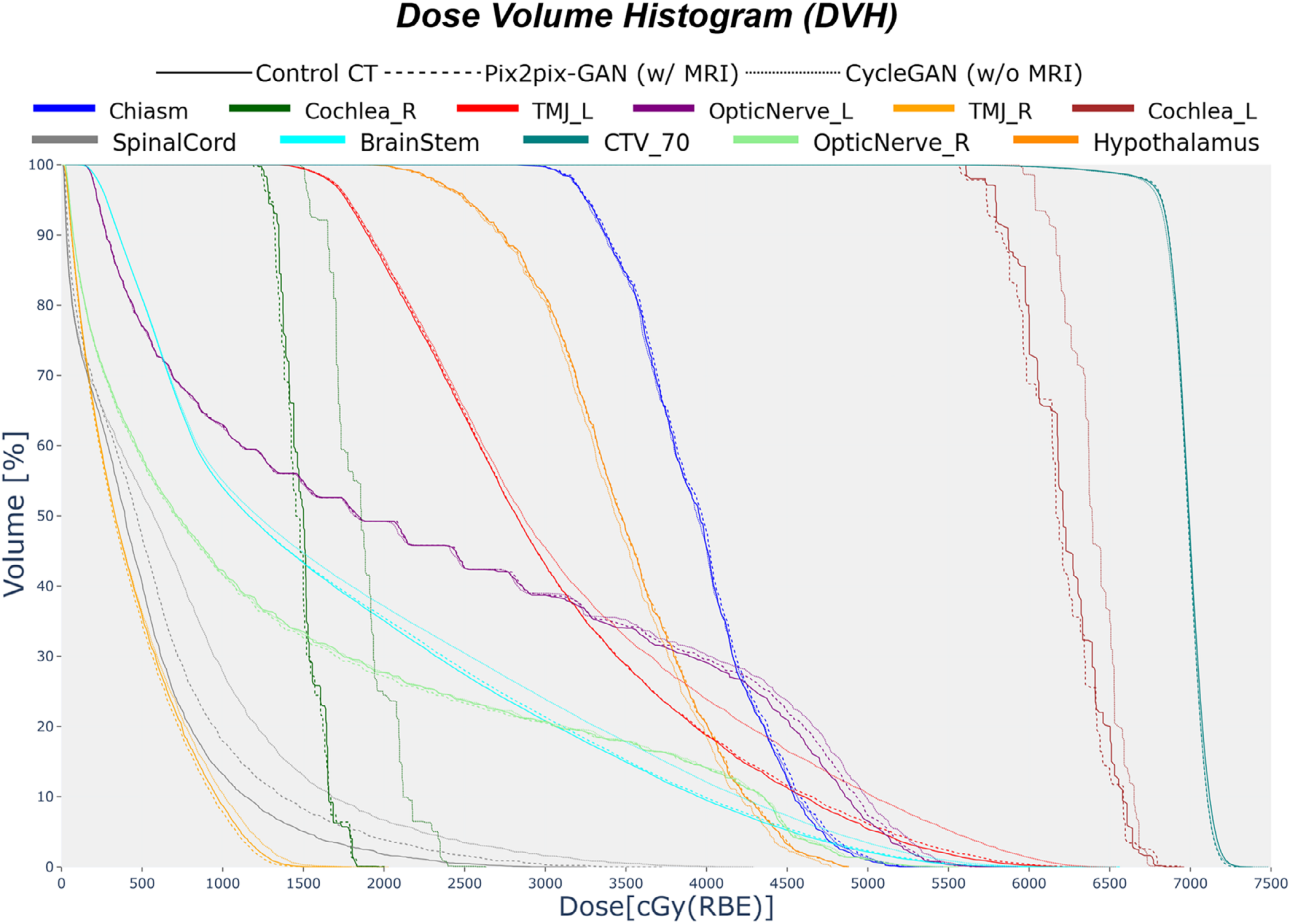
DVH is showcased for different OARs and CTV to assess our models' performance. Each color relates to one organ. Solid, dashed, and dotted lines correspond to the CT ground truth, the MR‐based model, and the non‐MR‐based model, respectively. CT, computed tomography; DVH, dose volume histograms; OARs, organs at risk.

The impact of these HU discrepancies is also evident within the DVH of the patients. An example is shown in Figure 6, which compares dose distributions obtained from the control CT and the sCTs for OARs and target volumes. The dose distributions derived from the MR‐based model (dashed lines) align much closer with those of the control CT (solid lines) when compared with the non‐MR‐based dose distributions (dotted lines), particularly for critical structures such as the brainstem, temporomandibular joint, spinal cord, and cochleas. This enhancement in the alignment accuracy minimizes potential deviations in dose delivery, ensuring that high‐priority regions are adequately protected. For high‐dose regions, such as the clinical target volume, MR‐based Pix2Pix‐GAN also demonstrates consistency with the control CT, emphasizing its reliability in preserving tumor coverage.

**FIGURE 7:**
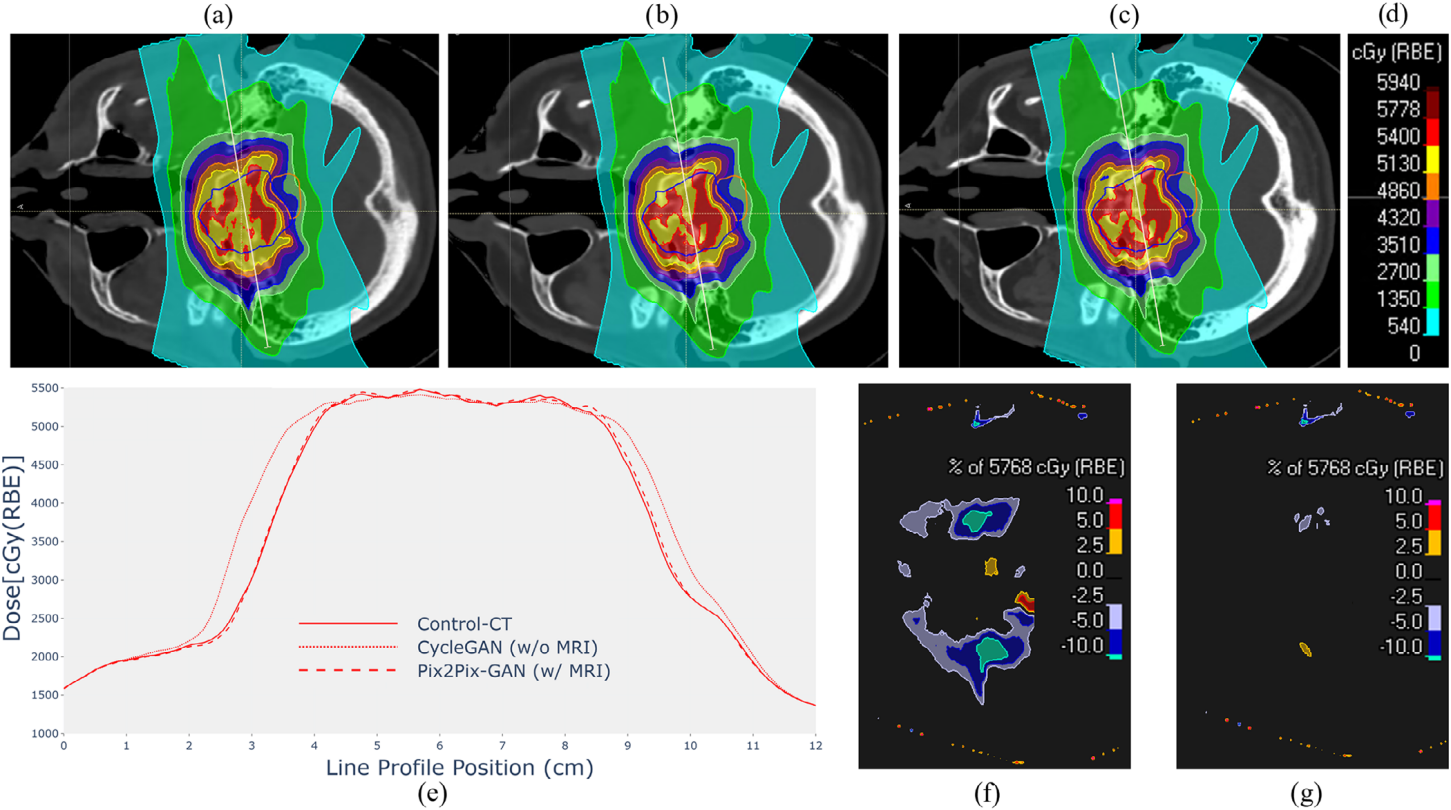
Dose distribution is shown on a specific slice for not MR‐based CycleGAN (a), MR‐based Pix2Pix‐GAN (b), and Control CT (c), with the color legend (d) of the dose values. Yellow line profiles showcased in those three top figures are compared in detail in the bottom chart (e). The map of dose differences compared to the Control CT is displayed for the non‐MR‐based model (f) and the MR‐based model (g). CT, computed tomography; CycleGAN, cycle‐consistent generative adversarial network; GAN, generative adversarial network.

The clinical viability of sCT was evaluated through dose calculation assessment comparing both MR‐based and non‐MR‐based approaches with those obtained using the control CT (Figure 7). The dose distribution of the CycleGAN model (Figure 7a) shows visible deviations compared to the Control CT (Figure 7c). This discrepancy suggests that relying solely on CBCT information limits the model's capacity to reproduce accurate tissue density representations, leading to suboptimal dose estimation. In the case of the Pix2Pix‐GAN (Figure 7b), the dose distribution is noticeably closer to that of the control CT. The incorporation of MRI as an additional input provides further anatomical detail, enabling more accurate tissue differentiation and resulting in a dose map that better aligns with the ground truth. When comparing dose profiles (Figure 7e) of CT ground truth (red solid line) and MR‐based model (red dashed line), there is practically no difference between them, unlike the approach without MRI (red dotted line). For the latter, the error diverges more significantly, particularly within regions of healthy tissue surrounding the target volume, which correspond to areas with steep dose gradients. The dose difference between sCTs and CT ground truth is also evaluated (Figure 7f,g). The magnitude and spread of the error are significantly reduced for the MR‐based model, following that MRI data improves the accuracy and reliability of dose calculation made with sCT volume.

**FIGURE 8:**
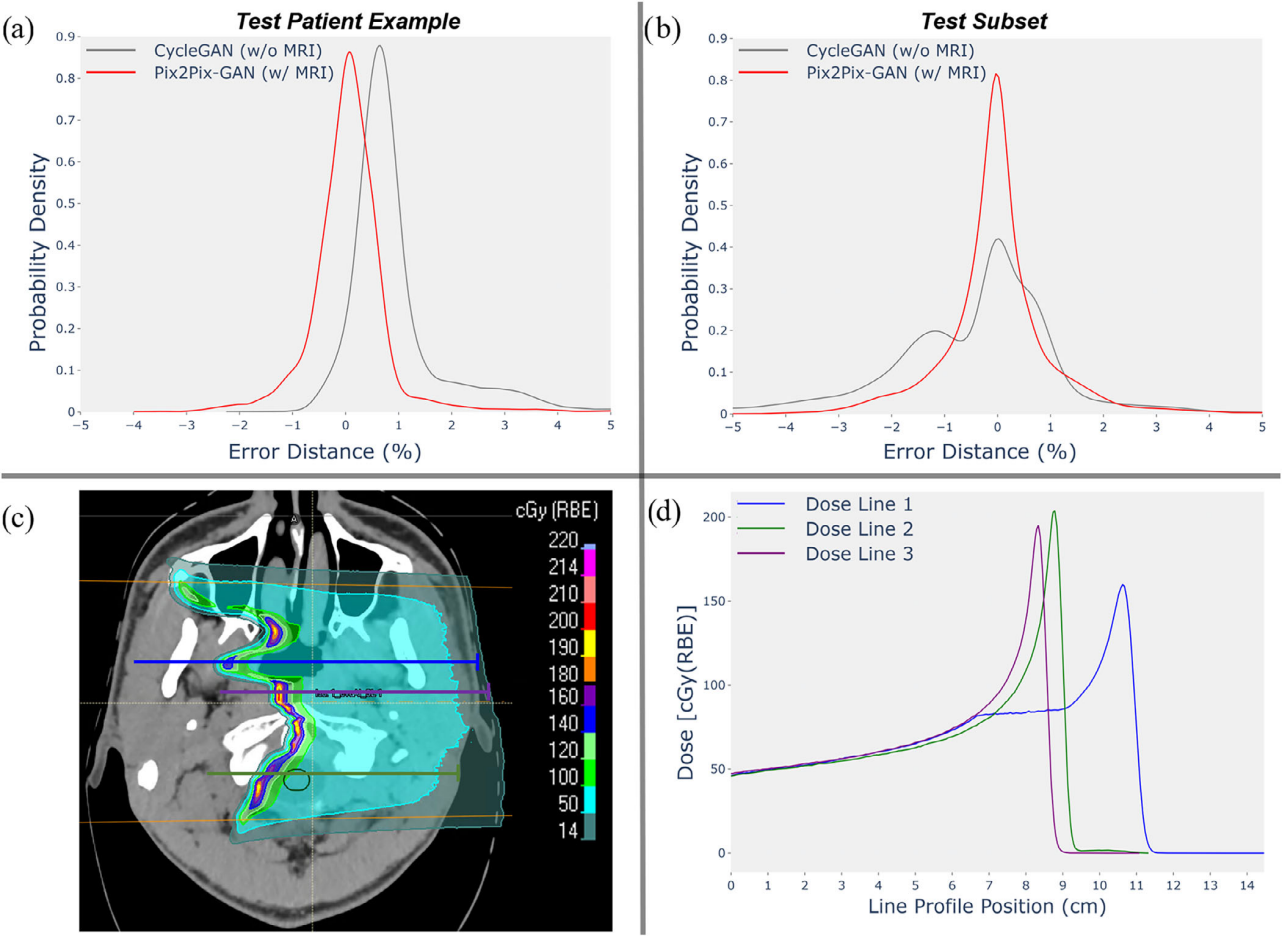
(a) Histogram of R80 distances difference (% error) is shown for a test patient example, and (b) for the whole test subset, analyzing MR‐based and non‐MR‐based sCTs. (c) Different line dose examples along the lateral direction were used to evaluate the distal R80 of a single‐energy lateral proton beam within the patient's anatomy, and (d) line profile position, corresponding to 1 × 1 ${\mathrm{mm}}^{2}$ beamlets. sCTs, synthetic CTs.

Figure 8 shows the distance error distribution with regard to distal R80 of a monoenergetic proton beam released within the patient's anatomy. Distal R80 was evaluated using dose lines within a specific volume. For illustrative purposes, each of the three dose line examples marked in the lateral direction of the patient's CT slice in Figure 8c corresponds to a dose profile in Figure 8d, each dose line having a different color to associate it with its corresponding dose profile. Once the R80 map is extracted for each image, the differences between both images (MR‐based Pix2Pix‐GAN versus non‐MR‐based CycleGAN) are assessed relative to the Control CT to obtain the histograms of R80 value differences. The distance at which the isodose lines disappear in the CT image (Figure 8c) indicates where the protons have stopped, representing the depth range they reach. The earlier the protons stop, the more the dose profile in Figure 8d will be shifted to the left lateral direction.

Histogram analysis shows for the test patient example (Figure 8a) and the whole test subset (Figure 8b) how the MR‐based model produces a narrower distribution of R80 errors with lower variability and a more concentrated error distribution around zero error percentage. This leads to better accuracy in estimating R80 values and a more reliable estimation of the proton range compared to the non‐MR‐based approach. The latter exhibits a wider and more dispersed error distribution, suggesting greater variability and potential inaccuracies in range prediction due to higher susceptibility to uncertainties. These discrepancies could lead to unintended dose deposition beyond the intended target, increasing the risk of underdosage in the tumor and overdosage in critical organs.

## 6 DISCUSSION

Qualitative observation (image visualization, axial HU profiles), quantitative evaluation (image metrics, OARs delineation metrics, HU error maps), and dosimetric analysis (DVH, dose profiles, dose distribution, R80 maps) enabled the assessment of sCT obtained. Quantitative and qualitative results of sCT images improve when incorporating MRI data in the CBCT‐based CT synthesis. Due to its superior image quality, MRI compensates for the lack of soft tissue definition in CBCT and allows for recovery of detail in organs like the eyes or the ventricles. With respect to architecture performance, SwinUnet should provide, in principle, better results than a classic ResUnet CNN, as the former can learn complex patterns and generalize better, but this does not happen in our case. SwinUnet‐based models underperformed ResUnet approaches, and this could occur due to our small database. VTs often need a large and diverse database to outperform CNN results. In addition, the neural network receives two simultaneous inputs (CBCT and MRI), requiring even more data to generalize correctly. Our Pix2Pix‐GAN provided superior qualitative results when compared to the autoencoder trained in isolation, as the competition between the discriminator and the generator increases the robustness of the generator/autoencoder. On a more detailed analysis, the axial profile revealed the crucial impact of MRI on the brain ventricles and ocular regions, allowing for following the CT original profile path and maintaining the high frequency and amplitude of the CT original signal. Although our Pix2Pix‐GAN complies with the task proposed, we also implemented a CycleGAN (w/o MRI) to compare the performance between both cGANs. Moreover, DL‐based segmentation on OARs was carried out using sCTs to evaluate the automatic contour delimitation improvement when using MRI as input.

Even though the impact of MRI is quite noticeable for individual proton beam dose distribution, it decreases with the combination of all beams together. This is because robust optimization (geometry, ballistics) used in dose planning mitigates HU to SPR conversion uncertainty in both target and OARs. Despite having no significant variations in the statistics of CTV, such a difference is concentrated within the surrounding healthy tissue. In situations where the tumor presents a cystic component, the use of MRI would be a differential advantage during dose planning due to its contribution to achieving tissue contrast on the brain ventricles. The non‐MR‐based model underestimates HU and SPR values, overestimating the dose delivered to surrounding healthy tissue as well as target dose coverage, which leads to underdose situations on the target volume. This error is about 1 mm around the target, which corresponds to 1% range uncertainty, consistent with HU errors. This hampers the clinical evaluation of the treatment because it can lead to wrong decisions about the need for plan adaptation.

Weekly CBCT‐CT pairs corresponding to the fractionated treatment PT sessions were also included, apart from CBCT‐CT pairs related to the initial treatment planning. The temporal distance between the planning MRI acquired at the beginning of treatment and the weekly CBCT‐CT pairs obtained throughout treatment is sometimes considerable, and this poses a challenge for neural network learning. However, this does not represent a limitation, as the neural network learns based on the current CBCT‐CT images that contain the updated information of the patient tissues. The fact that treatment planning remains accurate despite the temporal gap between the MRI and the CBCT‐CT pairs demonstrates the robustness of the model proposed.

In both training and test datasets, anatomical differences between CT‐CBCT image pairs are expected to be minimal, as these images are acquired within a short time frame. This holds true regardless of any anatomical differences between the CT‐CBCT pairs and the initial CT scan (used for treatment planning) and the MR image (acquired before the beginning of the treatment). Our primary objective was to evaluate the accuracy and uncertainty of the resulting sCT images when applied to contouring and dose calculation, as it becomes critical for their potential application in online adaptive workflows, where a reference image for adaptation would be acquired in the patient's treatment position and used for contouring and dose calculation, probably using automatic tools. Furthermore, since each patient has multiple CBCT‐CT image pairs acquired throughout the course of the treatment, our DL models have been able to be trained with tumors related to different treatment stages, making the proposed approach more robust and reliable.

Though valuable, this study does present some limitations. The complexity of collecting a large multimodal database limits the size and diversity of the dataset. Including a larger number of patients and a multi‐center database would enhance the reliability and robustness of the models. Further development may also be done with other MRI modalities to verify that sCT performance does not depend on a specific MRI sequence. It would also be advisable to adapt the MRI acquisition protocol in order to match both the number of slices and FOV with respect to CBCT‐CT image pairs. Another future line of work might be to evaluate replanning patient cases, also including other anatomical regions, in which significant anatomical changes will be observed, and they will require significant treatment adaptation.

## 7 CONCLUSION

Significant anatomical changes might occur during fractionated PT, especially for HC, potentially undermining the effectiveness of the treatment. This variability makes it essential to adapt the planned treatment, and the development of a highly automated process for generating sCT images represents a crucial advantage to improve the clinical workflow in APT. This work, following the continuation of our previous study,^8^ demonstrates that MRI data helps to upgrade both the recovery of soft tissue detail and dose precision when being incorporated within in‐room CBCT‐based CT synthesis. Thanks to the undertaken evaluation obtained with sCT images, we were able to prove how our MRI‐based models (particularly in the case of our Pix2Pix‐GAN‐ResUnet model) enhanced qualitative, quantitative, and dosimetric performance. Nonetheless, despite all these promising results, this work represents a first proof of concept, and additional studies need to be conducted. As a future goal, we aim to validate the proposed pipeline to further integrate the initial planning MRI into the CBCT‐based CT synthesis process so that we successfully optimize the APT's usual clinical workflow.

## AUTHOR CONTRIBUTIONS

*Conceptualization, study conception, and protocol design*: Juan Manuel Molina‐Maza, David Viar‐Hernande, Norberto Malpica, Angel Torrado‐Carvajal, and Blanca Rodriguez‐Gonzalez. *Data collection and preprocessing*: Juan Manuel Molina‐Maza, David Viar‐Hernandez, and Javier Sequeiro‐Gonzalez. *Algorithm design, training and software implementation*: Juan Manuel Molina‐Maza. *Evaluation and validation of results*: Juan Manuel Molina‐Maza, Blanca Rodriguez‐Gonzalez, and Juan Maria Perez‐Moreno. *Writing‐original draft preparation*: Juan Manuel Molina‐Maza. *Writing‐review and editing*: Juan Manuel Molina‐Maza, Angel Torrado‐Carvajal, Juan Maria Perez‐Moreno, and Borja Rodriguez‐Vila. *Project administration and funding acquisition*: Alejandro Maza, Juan Antonio Vera‐Sanchez, Norberto Malpica, Angel Torrado‐Carvajal, Juan Maria Perez‐Moreno, and Borja Rodriguez‐Vila. All authors have read and approved the final version of the manuscript.

## CONFLICT OF INTEREST STATEMENT

The authors declare no conflicts of interest.

## Figures and Tables

**FIGURE A.1:**
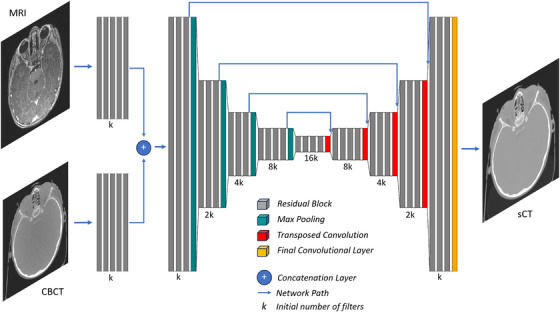
General overview of our ResUnet structure. It shows the case where CBCT and MRI inputs are both available. When we only have CBCT as input, the first initial stage, containing two groups of five residual blocks each (shown in the figure left), is not included in the autoencoder architecture. CBCT, cone‐beam computed tomography; MRI, magnetic resonance image.

**FIGURE A.2:**
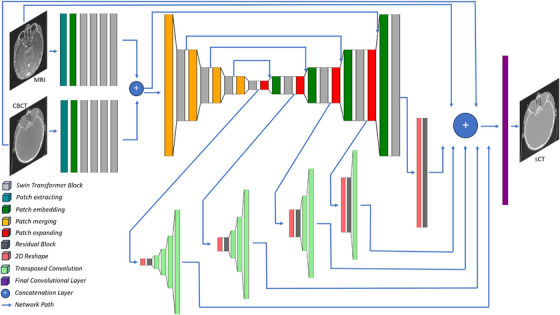
General overview of our SwinUnet structure. It shows the case where CBCT and MRI inputs are both available. When we only have CBCT as input, the first two initial stages containing four ST blocks each (shown in the figure left) are not included in the autoencoder architecture. CBCT, cone‐beam computed tomography; MRI, magnetic resonance image.

**FIGURE A.3:**
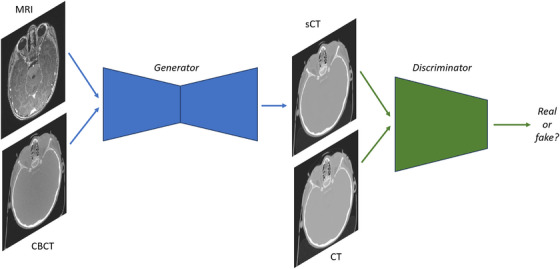
General overview of our Pix2Pix cGAN structure. The generator processes both CBCT and MRI inputs and synthesizes an sCT. Then, sCT and CT are fed into the discriminator to decide if the sCT image obtained is real or fake. CT, computed tomography; CBCT, cone‐beam CT; GAN, generative adversarial network; MRI, magnetic resonance image; sCTs, synthetic CTs.

**FIGURE A.4:**
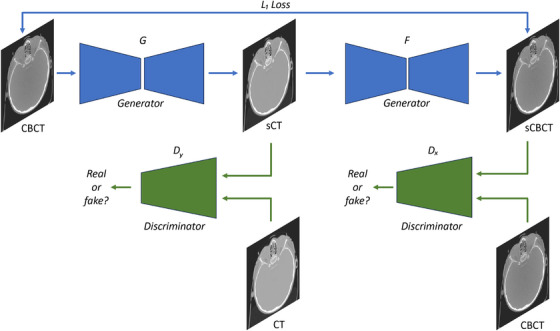
General overview of our CycleGAN structure. Generator G goes from CBCT to CT, and generator F does the opposite transformation. Discriminators $D_{x}$ and $D_{y}$ try to differentiate between synthetic and real images. CT, computed tomography; CBCT, cone‐beam CT; CycleGAN, cycle‐consistent generative adversarial network.

## APPENDIX A

### A.1 ResUnet

We define the Residual‐Unet or ResUnet (Figure A.1) as a classical U‐Net architecture^42^ where residual blocks and skip connections are added. The proposed model relies on four encoding and four decoding stages, and the last convolutional layer is activated by a linear function. Skip connections concatenate feature maps from the encoding and decoding stages, and residual blocks are incorporated inside each stage, including two 2D convolutions (kernel size = 3 × 3), two normalization layers, and two ReLu activation functions. The encoding/decoding stages reduce/increase the image size and increase/reduce the complexity of the maps. The initial number of filters is 64 and is doubled/halved for encoding/decoding stages, where max‐pooling/transposed convolutions operations are used for downsampling/upsampling. When we feed our ResUnet with both CBCT and MRI inputs, there is an initial stage where each input is first processed with 5 residual blocks, and then we concatenate the feature maps resulting from every input. This way, the neural network first learns each input independently before combining its information at the end of the stage.

### A.2 SwinUnet

SwinUnet design proposed by Liu et al.^37^ was chosen as the basic structure for our neural network (Figure A.2), where eight transformer stages were implemented (four encoding stages and four decoding stages) and ST blocks (window size = 4, MLP = 512), following the original implementation. First, a patch‐extracting layer splits the input slice into non‐overlapping 4 × 4 patches, and then a patch‐embedding layer linearly projects the initial channels to 64. All encoding and decoding stages have one ST block, except for the first encoding stage, which contains four consecutive ST blocks (if both inputs provided are concatenated before the first patch merging). For every encoder/decoder stage, a patch merging/patch‐expanding layer reduces/increases the number of patches by half/double and duplicates/halves the channel dimension. Feature maps from the encoding and decoding phases are connected through skip connections. In the case of decoding stages, patch embedding layers keep the consistency in the number of channels by halving them after the skip connections. A convolutional decoder phase was also implemented to help recover the spatial dimension of the original images. Transformer feature maps obtained after each ST block in the decoder were reshaped into 2D maps, processed with a convolutional residual block, and fed into consecutive transposed convolutions. Finally, a concatenation layer fused all the information received from input images and the outputs of final transposed convolutions. A final convolutional layer (kernel size = 1 × 1, linear activation function) got the output of this concatenation layer and generated the final sCT slice.

### A.3 Pix2Pix‐conditional GAN

Among image cGANs, we chose Pix2pix‐GAN^35^ because both CBCT and MRI were our simultaneous inputs, and it would be difficult for the CycleGAN to retrieve the information in the inverse generator, that is, converting a single CT slice into CBCT and MRI slices. Pix2pix approach has also demonstrated superior results for brain medical image synthesis compared to CycleGAN when dealing with registered data.^43^ We did not add any z random noise to the input image, as the generator G could just ignore the noise and fool the discriminator D easily. The loss function of our Pix2Pix cGAN can be articulated as follows:

(A.1)
$$L_{cGAN}\left(G,D\right)=E_{x,y}\left[\log D(x,y)\right]+E_{x}\left[\log1-D(x,G(x))\right]$$

We use L1 distance as a regularizer (λ = 1) to force the generator not only to mislead the discriminator but also to stay near the ground truth in an L1 manner:

(A.2)
$$L_{L1}\left(G\right)=E_{x,y}{[∥y-G\left(x\right)∥}_{1}]$$

The generator G aims to minimize such loss function competing against the discriminator D, being the objective function to achieve:

(A.3)
$$G^{*}=\arg\min_{G}\max_{D}L_{cGAN}\left(G,D\right)+\lambda L_{L1}\left(G\right)$$

ResUnet (Figure A.1) and SwinUnet (Figure A.2) GAN generators were implemented and compared. The GAN discriminator only focuses on high‐frequency details, as the L1 loss term ensures accurate low‐frequency representation. Instead of analyzing the entire image, we use a PatchGAN discriminator to classify each small N × N image patch as real or fake,^35^ and then average the responses of all patch classifiers to get the final discriminator output (in our case, 64 × 64 final patches). The discriminator architecture is based on the ResUnet encoder: four encoder stages and three max‐pooling operations. After the fourth encoder stage, a convolutional layer (kernel size = 1 × 1) is applied to get the final 64 × 64 × 1 shape. To summarize, Figure A.3 depicts our Pix2Pix cGAN scheme.

### A.4 Cycle‐consistent generative adversarial network

The CycleGAN framework^36^ operates through two interconnected generative networks – G and F– that perform translations in opposite directions between two domains, X (CBCT) and Y (CT). Specifically, the generator G maps images from X to Y, while the generator F reverses the process. To evaluate the quality of these mappings, two discriminators – $D_{X}$ and $D_{Y}$ – are introduced. $D_{X}$ differentiates between real and synthesized images in X, while $D_{Y}$ performs the same for Y domain. Figure A.4 presents our CycleGAN architecture. Generators G and F both follow our ResUnet design, and discriminators $D_{X}$ and $D_{Y}$ are built with a patch‐based approach. Several loss functions guide the networks toward generating high‐quality translations:

- **Adversarial Loss**. This loss ensures the realistic appearance of both sCT and sCBCT. For the generator G to map X→Y (a similar term applies to the reverse mapping F:Y→X):
  (A.4)
  $$\begin{array}{cc} & L_{GAN}\left(G,D_{Y},X,Y\right)\\ & \;=E_{y∼p_{data}\left(y\right)}\left[\log D_{Y}\left(y\right)\right]+E_{x∼p_{data}\left(x\right)}\left[\log\left(1-D_{Y}\left(G\left(x\right)\right)\right)\right]. \end{array}$$
- **Cycle Consistency Loss**. This loss enforces consistency between input and output images when they undergo translation in both directions, ensuring that translating an image and then reversing the process reproduces the original input:
  (A.5)
  $$\begin{array}{cc} L_{cycle}\left(G,F\right) & =E_{x∼p_{data}\left(x\right)}{[∥F\left(G\left(x\right)\right)-x∥}_{1}]\\ & \;+E_{y∼p_{data}\left(y\right)}{[∥G\left(F\left(y\right)\right)-y∥}_{1}]. \end{array}$$
- **Combined Objective Loss**. It integrates both the adversarial and cycle consistency losses, with a weighting parameter λ:
  (A.6)
  $$\begin{array}{cc} L(G,F,D_{X},D_{Y}) & =L_{GAN}\left(G,D_{Y},X,Y\right)\\ & \;+L_{GAN}\left(F,D_{X},Y,X\right)+\lambda L_{cycle}\left(G,F\right). \end{array}$$

### A.5 Delineation metrics

#### A.5.1 Dice similarity coefficient

DSC measures the overlap between the predicted and the ground truth segmentation to evaluate their similarity. It is calculated as the ratio of twice the area of overlap between the prediction and ground truth to the total number of pixels in both sets:

(A.7)
$$\text{DSC}=\frac{2|A\cap B|}{\left|A|+|B\right|}$$

where A and B represent the prediction and the ground truth, respectively. DSC ranges from 0 to 1, where 0 indicates no overlap and 1 signifies a perfect overlap. DSC takes into account both false positives (pixels incorrectly included in the predicted segmentation) and false negatives (pixels that are missed), but it does not account for spatial distribution or shape differences, necessitating complementary metrics such as the HD.^40^, ^41^

#### A.5.2 Hausdorff distance

HD evaluates the spatial similarity between the predicted and the ground truth boundary by calculating the maximum distance of a point in one set to its nearest neighbor in the other set. It identifies the point on one boundary that is farthest from the other boundary and measures the distance to the closest point on the other boundary:

(A.8)
$$h\left(A,B\right)=\max_{a\in A}\min_{b\in B}∥a-b∥$$

where ∥a−b∥ represents the Euclidean distance between a point a in set A and a point b in set B. To ensure that the metric gets the largest possible deviation in either direction, the bidirectional HD is defined as:

(A.9)
H(A,B)=max{h(A,B),h(B,A)}

HD is very sensitive to outliers, as it identifies the point of maximum error between the two sets. A small region of significant boundary deviation will lead to a high HD value. This makes HD a strict and informative metric for PT planning, where small delineation errors can have significant implications. Nevertheless, a single outlier or minor misalignment can disproportionately increase the HD, even if the majority of the segmentation aligns well with the ground truth. Therefore, the HD is often used in combination with other metrics, such as the DSC.^40^, ^41^
